# Design of Wearable Headset with Steady State Visually Evoked Potential-Based Brain Computer Interface

**DOI:** 10.3390/mi10100681

**Published:** 2019-10-10

**Authors:** Bor-Shyh Lin, Bor-Shing Lin, Tzu-Hsiang Yen, Chien-Chin Hsu, Yao-Chin Wang

**Affiliations:** 1 Institute of Imaging and Biomedical Photonics, National Chiao Tung University, Hsinchu City 30010, Taiwan; 2 Department of Computer Science and Information Engineering, National Taipei University, New Taipei City 23741, Taiwan; 3 Department of Emergency Medicine, Chi Mei Medical Center, Tainan City 71004, Taiwan; 4 Department of Computer Science and Information Engineering, Cheng Shiu University, Kaohsiung City 83347, Taiwan

**Keywords:** brain–computer interface (BCI), steady-state visually evoked potentials (SSVEP), field-programmable gate array (FPGA), wearable

## Abstract

Brain–computer interface (BCI) is a system that allows people to communicate directly with external machines via recognizing brain activities without manual operation. However, for most current BCI systems, conventional electroencephalography (EEG) machines and computers are usually required to acquire EEG signal and translate them into control commands, respectively. The sizes of the above machines are usually large, and this increases the limitation for daily applications. Moreover, conventional EEG electrodes also require conductive gels to improve the EEG signal quality. This causes discomfort and inconvenience of use, while the conductive gels may also encounter the problem of drying out during prolonged measurements. In order to improve the above issues, a wearable headset with steady-state visually evoked potential (SSVEP)-based BCI is proposed in this study. Active dry electrodes were designed and implemented to acquire a good EEG signal quality without conductive gels from the hairy site. The SSVEP BCI algorithm was also implemented into the designed field-programmable gate array (FPGA)-based BCI module to translate SSVEP signals into control commands in real time. Moreover, a commercial tablet was used as the visual stimulus device to provide graphic control icons. The whole system was designed as a wearable device to improve convenience of use in daily life, and it could acquire and translate EEG signal directly in the front-end headset. Finally, the performance of the proposed system was validated, and the results showed that it had excellent performance (information transfer rate = 36.08 bits/min).

## 1. Introduction

Brain–computer interface (BCI) is a system that allows people to communicate directly with external machines via brain activity signals [1,2,3,4]. Being able to communicate with external machines without any physical action is useful for severely disabled people [5]. In general, electroencephalography (EEG) is the most frequently used brain activity signal in BCI applications due to its properties of lower cost, noninvasive measurement, and higher time resolution. In previous studies, several types of EEG signals, including visual evoked potential (VEP) [6], slow cortical potentials [7], μ rhythm related to motor imagery (MI) [8], and P300 evoked potentials [9], have been successfully used as the control command of BCI. Steady-state visually evoked potential (SSVEP) is one of the most frequently used methods in BCI applications due its high information transfer rate (ITR) [10], convenience and ease of use, and lower training requirement [11,12,13].

In 1997, Skidmore et al. found that EEG spectrum obtained from the main visual cortex could present stronger peaks at visual stimulus frequencies, and this system was certified using functional magnetic resonance imaging (FMRI) [14]. In 2002, Cheng et al. proposed a SSVEP-based BCI system with 12 visual stimulus signals, and the user could dial a phone number by only focusing on flags of different flicker frequencies on the computer screen [15]. However, the above system used a computer screen as the visual stimulus device, and its large size and weight meant it had poor portability.

In 2008, Muller-Putz et al. designed a SSVEP-based BCI system to control an electrical hand prosthesis, and light-emitting diodes (LEDs) were also used for the design of a visual stimulus device to replace the computer screen [16]. Here, visual stimuli sources with the frequencies of 6, 7, 8, and 13 Hz were used to control wrist rotation to the left, wrist rotation to the right, opening of the fingers, and closing of the fingers of the electrical hand prosthesis, respectively. Most of the above SSVEP-based BCI systems require a conventional EEG machine and EEG electrodes with conductive gels to acquire SSVEP signals. However, the large size of conventional EEG machines and the requirement of EEG leads to connect with EEG electrode causes inconvenience of use in daily life. Moreover, conductive gels usually encounter the problem of drying out and hardening during prolonged measurements. Di Flumeri et al. tested different dry and gel-based systems [17]. It was possible to obtain results comparable to wet electrodes in terms of signals spectra and classification of mental states, but it drastically reduced the time of montage and the comfort level. In particular, multiple-pin and solid-gel electrodes are better than gold-coated, single pin-based ones in terms of comfort.

In 2011, Wang et al. developed a dry electrode for measuring SSVEP signals to improve the drying issue of conventional EEG electrodes with conductive gels [18]. Here, a commercial personal computer was used as the back-end digital signal processing platform. However, using a personal computer as the back-end BCI platform is still inconvenient for use in daily life due to its large size and weight. In 2018, Lin et al. proposed a SSVEP BCI system that used phase coding of SSVEP as the control command of BCI. The BCI algorithm was also implemented in field-programmable gate array (FPGA) to reduce the size of the back-end BCI platform and improve convenience of use [19]. Here, conventional EEG electrodes with conductive gels were still used to acquire SSVEP. They also indicated that the ITR of the phase coding method was relatively lower than that of the frequency coding method.

In order to improve the issues of previous SSVEP-based BCIs, a wearable headset with SSVEP-based BCI was designed and implemented in this study. Here, a novel active dry electrode was designed to acquire SSVEP in hairy site without conductive gels. Moreover, the BCI algorithm was implemented in FPGA to reduce the size of the back-end BCI platform. Both the active dry electrodes and the BCI platform were embedded in a wearable mechanical design to acquire and translate EEG signals directly. Moreover, instead of the use of LEDs as the source of visual stimulus as in previous SSVEP-based BCIs, a commercial tablet was used as a visual stimulus device to provide control graphic icons. Finally, the performance of the proposed wearable SSVEP-based BCI in acquiring SSVEP signal quality and information transfer rate were investigated and validated. The experimental results showed that the proposed wearable SSVEP-based BCI could effectively acquire SSVEP in hairy site without conductive gels and provide a good ITR. Therefore, the proposed system can be viewed as a good prototype of wearable BCI, and it may be applied to many daily life applications in the future. 

## 2. System Architecture and Implementation

**Figure 1:**
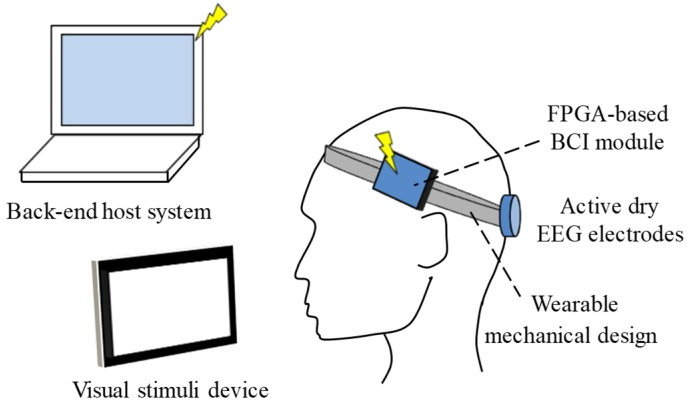
Basic scheme of proposed wearable headset with steady-state visually evoked potential (SSVEP)-based brain–computer interface (BCI).

SSVEPs are natural, physiological feedback signals generated by an individual’s brain when their eyes receive a visual stimulus with a specific frequency that is higher than 6 Hz [20,21]. The generated SSVEP also provides a great contribution at the specific frequency in the EEG spectrum. SSVEP can be used as a mental control method in BCI systems. The basic scheme of the proposed wearable headset with SSVEP-based BCI is shown in Figure 1. It mainly contains active dry EEG electrodes, a wearable mechanical design, FPGA-based BCI module, and a visual stimulus device. The visual stimulus device was designed to generate visual stimuli with specific frequencies. The active dry electrodes were designed to acquire SSVEP without conduction gels in hairy site from the location of Oz in 10–20 EEG system. The obtained SSVEP would then be amplified and processed by the FPGA-based BCI module to extract the specific frequency of SSVEP and transmit the information to the back-end host system. Fast Fourier transform (FFT) was implemented in the FPGA-based BCI module to extract the SSVEP spectrum. The back-end host system would determine the control command according to the frequency information of SSVEP obtained by the proposed system to control the external device. The use of the wearable mechanical design was aimed at allowing the user to more easily wear and take off the proposed system. 

### 2.1. Active Dry EEG Electrodes and Wearable Mechanical Design

**Figure 2:**
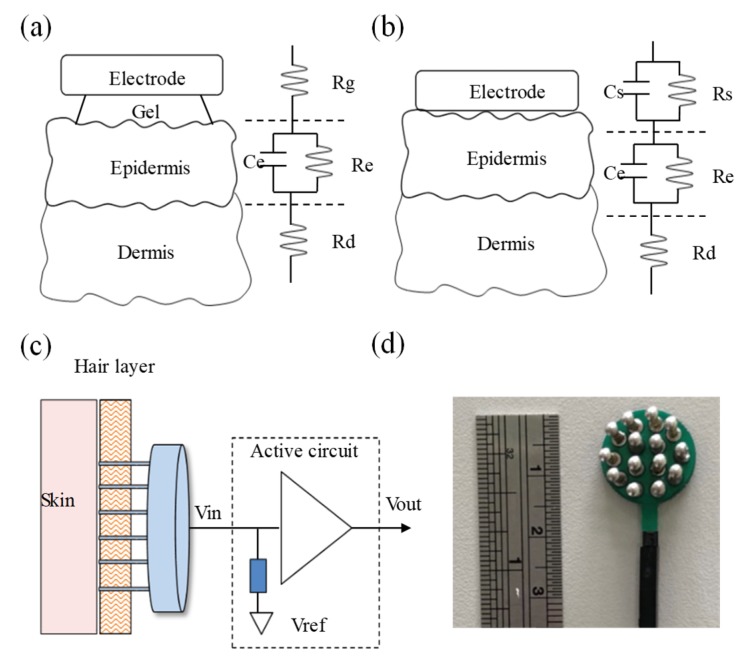
Skin–electrode interface models of (**a**) electroencephalography (EEG) electrode with conductive gels and (**b**) dry electrode. (**c**) Basic scheme and (**d**) photograph of active dry electrode.

In general, conventional EEG electrodes with conductive gels (wet electrode) [22] are used to measure EEG. However, wet electrodes can easily encounter the problem of drying out. In recent years, several dry electrodes have been proposed to measure biopotentials without conductive gels to improve the above issue [23]. Figure 2a,b shows the skin–electrode interface models of the wet electrode and dry electrode, respectively. In order to effectively reduce the epidermis impedance to avoid the attenuation of the measured biopotentials, conductive gels are usually used to fill the space of the skin–electrode interface to transfer biopotentials from the epidermis layer to the high-ion conductive layer. Here, the equivalent circuit model of the conductive gel layer can be viewed as a resistance Rg connecting to the epidermis layer in series. The equivalent circuit model of the epidermis layer can be viewed as a resistance Re and a capacitor Ce in parallel, and it connects to the resistance model Rd of the dermis layer in series. The skin–electrode interface model of the dry electrode lacks a conductive gel layer, and the metal electrode has to directly contact with the skin. However, because the surface of the skin is usually not smooth, it is difficult for the dry electrode to completely and perfectly contact the skin. Therefore, the skin–electrode interface model contains the resistance Rs and the capacitor Cs characteristics [23,24]. Moreover, the overall skin–electrode interface impedance of the dry electrode is often greater than that of the wet electrode. In order to reduce the skin–electrode interface impedance of the dry electrode, an active dry electrode was designed and implemented, as shown in Figure 2c. It mainly contains a comb-shaped metal electrode and an active circuit. The comb-shaped metal electrode was made of copper to pass through the hairy layer to contact with the skin directly. Although the comb-shaped metal electrode could contact with the skin directly, its contacting area was still small to cause higher skin–electrode interface impedance. Therefore, an active circuit was designed to provide an ultra-high input impedance to avoid signal attenuation and phase distortion and reduce the influence of the common-mode rejection ratio. Figure 2d shows a photograph of the implemented active dry electrode.

**Figure 3:**
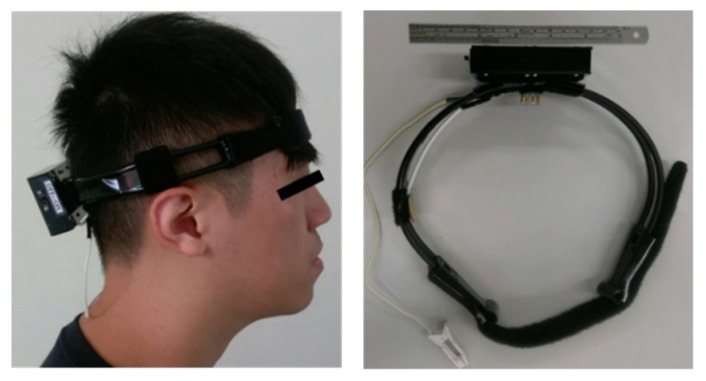
Photograph of wearable mechanical design in the proposed system.

In this study, a wearable mechanical design was also implemented to allow the user to easily wear or take off the proposed system, as shown in Figure 3. Moreover, the wearable mechanical design would also provide a suitable pressure to maintain a stable skin–electrode contacting condition.

### 2.2. FPGA-Based BCI Module

**Figure 4:**
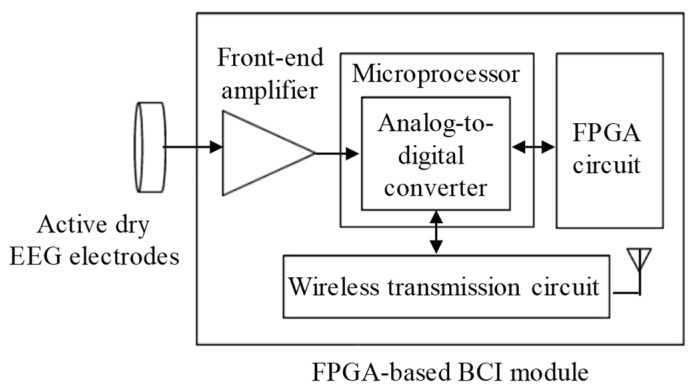
Block diagram of field-programmable gate array (FPGA)-based SSVEP BCI module.

The block diagram of the proposed FPGA-based BCI module is shown in Figure 4, and it mainly contains several parts, including a front-end amplifier, a microprocessor, a FPGA circuit, and a wireless transmission circuit. The signal of SSVEP would be first obtained by the proposed active dry EEG electrodes, and it would then be amplified and filtered by the front-end amplifier. The total gain of the front-end amplifier was set to 5000 v/v with frequency band of 0–40 Hz. Next, the amplified SSVEP signal would be digitized with sampling rate of 64 Hz by a 12-bit analog-to-digital converter built in the used microprocessor and then sent to the FPGA circuit to calculate the SSVEP spectrum. The FPGA circuit mainly contains the parts of random access memory (RAM) and digital signal processing (DSP). The SSVEP signal received from the microprocessor would be stored in RAM, and the DSP part would then perform the FFT algorithm. Here, the verilog hardware description language (verilog HDL) was designed via the software Vivado Design Suite to execute logic synthesis in the FPGA circuit. From the SSVEP spectrum, the control command would be determined and sent to the wireless transmission circuit. The wireless transmission unit circuit contains a printed circuit board antenna and a Bluetooth module with the Bluetooth v2.0+ EDR specification. Finally, the signals and spectra of SSVEP and control commands would be transmitted to the back-end host system wirelessly via the wireless transmission circuit.

### 2.3. Visual Stimulus Device

**Figure 5:**
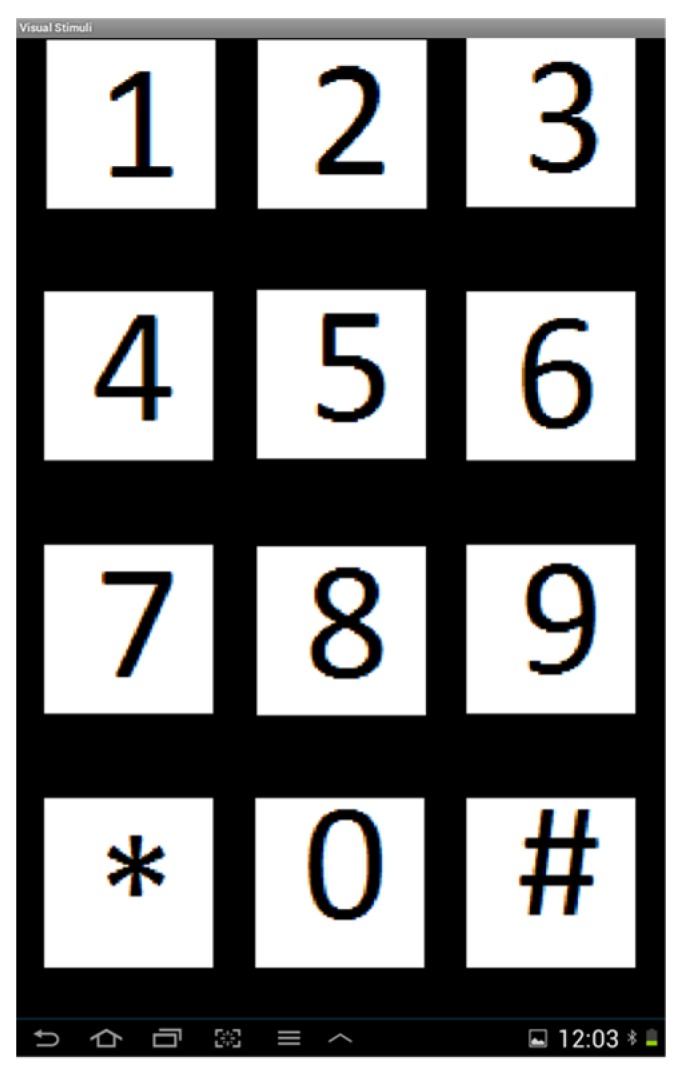
Screenshot of visual stimulus device.

In this study, a commercial tablet with the operating system Android 4.1.2 was used as a visual stimulus device. A total of 12 graphical icons with different specific flashing frequencies on the screen of the tablet were used as the source of visual stimuli, as shown in Figure 5. The frequency range of these flashing icons was from 8.5 to 14 Hz, and the frequency interval between the two nearest flashing icons was set to 0.5 Hz.

### 2.4. Implementation of SSVEP BCI Algorithm in FPGA

**Figure 6:**
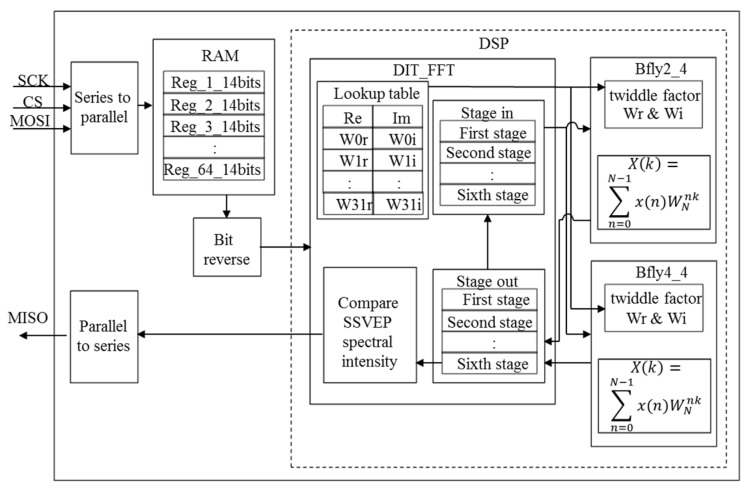
Hardware architecture of SSVEP BCI algorithm in FPGA.

The hardware architecture of SSVEP BCI algorithm in FPGA is shown in Figure 6. Here, Xilinx’s FPGA chip (Artix-7 Xc7a15tcsg324-1 FPGA, Xilinx, San José, CA, USA) with the working frequency of 80 MHz was connected with the microprocessor (RX210, Renesas, Tokyo, Japan) via series peripheral interface (SPI) protocol. First, the microprocessor would transmit a series data packet (64 SSVEP data with 32 overlap data) to the FPGA chip via master out slave in (MOSI), and the SSVEP data received would then be converted to the parallel data by the series-to-parallel module to facilitate the inside operation in the FPGA chip. Next, these SSVEP data would be stored in 64 registers (SSVEP1, SSVEP2,…, SSVEP64). In order to reduce the computational complexity of FFT, the presetting twiddle factor lookup table would also be used in the DIT_FFT module [24,25]. After bit reverse, the SSVEP data would be stored to the first stage of the DIT_FFT module and then sent to the butterfly operation module bfly2_4 or bfly4_4. If the input value of the butterfly operation module was only the real part or the imaginary part, then it would be sent to module bfly2_4; otherwise, if the module input value was complex, then it would be sent to module bfly4_4. The output of the butterfly operation module with the input of the first stage would be stored to the second stage of the DIT_FFT module and then sent to the butterfly operation module again. The above procedure would be repeated until the sixth stage value was obtained. The sixth stage value of the DIT_FFT module could be viewed as the output of FFT. Next, we extracted the FFT value corresponding to the specific frequencies of visual stimuli and then found the maximum value from the above extracted FFT values. The maximum FFT value corresponding to the specific frequencies of visual stimuli would then be sent to the parallel-to-series module to convert as a series data packet, and the series data packet would be transmitted to the microprocessor via MISO.

**Figure 7:**
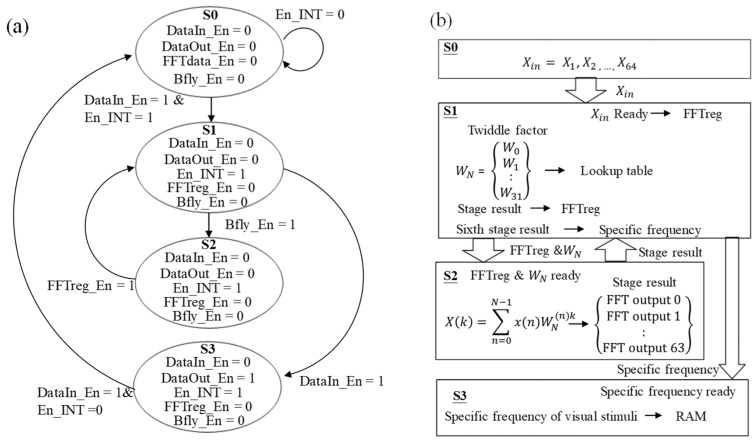
(**a**) State machine and (**b**) data flow of SSVEP BCI.

The finite state machine (FSM) and data flow of the SSVEP BCI algorithm in FPGA are shown in Figure 7a,b. The state machine of this system contains four states and five control signals, including DataIn_En, DataOut_En, En_INT, Bfly_En, and FFTreg_En. Here, DataIn_En was used to confirm whether to send data to the next stage. En_INT was designed to confirm whether to receive new data from the microprocessor. When 64 new data were received, it would become high to prevent the next new data from being received. FFTreg_En was used to store the current output of the butterfly operation when it was high. Bfly_En was used to confirm whether the currently register data was performed by the butterfly operation. After the complete FFT calculation, the specific frequencies would be obtained, and Bfly_En and FFTdata_En would be set to low and DataIn_En would be set to high to send them to the next state. DataOut_En was used to confirm whether the whole calculation had been completed and then send the result to the shift register and output the result when it became high. The whole procedure of SSVEP BCI algorithm in FPGA would be completed for four state cycles. In state S0, the SSVEP data would be received and then stored to RAM via a first-in-first-out (FIFO) register. After receiving 64 new data, EN_INT would become high and then perform state S1. In state S1, the received data would first be performed by bit reverse. Then, Bfly_En would become high to send these 64 data and the twiddle factors in the lookup table to the butterfly operation module bfly2_4 or bfly4_4 in state S2. After the butterfly operation module, FFTreg_En would become high to store the output of state S2 to the registers in state S1, and Bfly_En would then become high to resend the current output of state S2 into state S2. The above procedures would be executed six times to complete the whole FFT calculation. After the FFT calculation, the specific frequencies of visual stimuli could be obtained from FFT and compared, and both FFTreg_En and Bfly_En would become low and DataIn_En would become high to perform state S3. Finally, in state S3, the calculated specific frequency data of visual stimuli would be stored in RAM and sent to the microprocessor by the shift register. When the whole calculation has been completed, DataOut_En would become high to return to state S0. In this study, 6462 lookup tables (LUT), 12,238 flip flops (FF), five global clock buffers (BUFG), 13 digital signal processors (DSP), one mixed-mode clock manager (MMCM), and six input and output (IO) were used to implement the system. A total of 12 multipliers, eight adders, and five subtractors were used in this algorithm.

## 3. Results

### 3.1. Performance of Proposed System on Detecting SSVEP 

**Figure 8:**
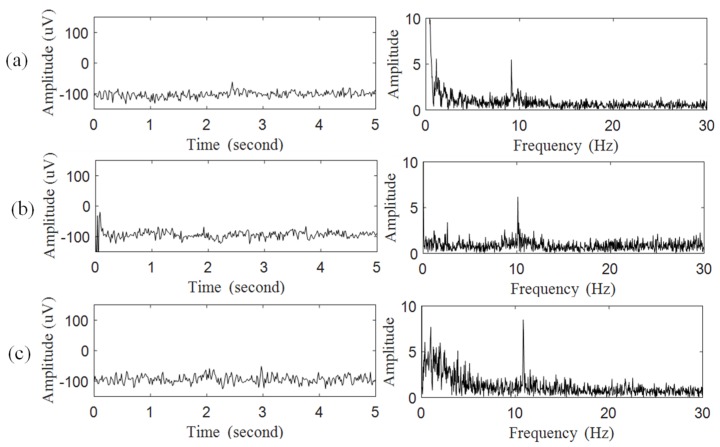
Raw SSVEP and their spectrum obtained by proposed BCI system when focusing on flashing icon with specific frequencies of (**a**) 9 Hz, (**b**) 10 Hz, and (**c**) 11 Hz.

The performance of the proposed system on the measurement of SSVEP was investigated. According to the international 10–20 EEG system [26], two dry electrodes were placed at T10 and Oz (primary visual cortex) as the reference and primary inputs to acquire SSVEP. During this experiment, the subject was instructed to focus on the flashing icons with the frequencies of 9, 10, and 11 Hz, respectively. The raw signals of SSVEP and their spectra are shown in Figure 8a–c, respectively. The experimental results showed the peaks in the EEG spectra accurately reflected the specific frequencies of these flashing icons.

### 3.2. Information Transfer Rate of Proposed System

The BCI performance of the proposed system, including the accuracy and ITR, was investigated. In this experiment, the subject was instructed to randomly focus on different flashing icons. A total of 20 subjects attended this experiment. Before evaluating the performance of the proposed system, several parameters for classification test were first defined as follows: true positive (TP) denoted that the recognized command was correctly identified as the target command; false positive (FP) denoted that the idle state was incorrectly identified as the other command; true negative (TN) denoted that the idle state was correctly identified as the idle state; and false negative (FN) denoted that the recognized command was incorrectly identified as the idle state or other command. Here, F-measure was used to evaluate the classification performance [27], and it can be calculated as follows:

(1) $$F\_measure=2\times \frac{precision\times recall}{precision+recall}\times 100\%$$

where precision is the positive predictive value (PPV), recall is the sensitivity, and they can be calculated as follows:

(2) $$precision=\frac{TP}{TP+FP}\times 100\%$$

(3) $$recall=\frac{TP}{TP+FN}\times 100\%$$

The experimental results showed that the averaged PPV, sensitivity, F-measure, and accuracy were about 96.33%, 95.83%, 95.96%, and 92.5%, respectively. Here, the definition of accuracy is given as follows:

(4) $$Accuracy\;=\frac{TP+TN}{TP+TN+FP+FN}\times 100\%$$

Next, the information transfer rate is an indicator widely used to evaluate the performance of the communication and control for BCI systems [28,29], and it can be given by

(5) $$B=\left[\log_{2}N+P\;\log_{2}\;P+\left(1-P\right)\log_{2}\left(\frac{1-P}{N-1}\right)\right]\left(\frac{60}{CTI}\right)$$

where N is the total number of control commands, P is the probability of correct selection, and *CTI* is the translation time for each command. In this study, the proposed system contained 12 control commands, and therefore *N* was equal to 12. The value of *CTI* was about 5 s. Therefore, the averaged ITR of the proposed system was about 36.08 (bits/min).

## 4. Discussions

Several SSVEP-based BCI systems have been designed in previous studies, and a comparison between the proposed BCI system and other SSVEP-based BCI systems is summarized in Table 1. Feng et al. designed a multimodal EEG-based hybrid BCI system to control robot movement and execute the grasp task [30]. Here, a back-end computer was designed to process EEG data and then generate the control command to control the external device. The FFT algorithm was used to extract the SSVEP spectral features to control the direction of travel of the robot. The motor imagery signal was designed to allow the robot to grab objects. In this system, the conventional EEG cap with conductive gels was used to acquire EEG signals. The portability and the convenience of long-term use for this system were limited. Wang et al. designed a wearable SSVEP-based BCI system to control a quadcopter [31]. Here, saline-based electrodes were used to acquire EEG signals without conductive gels, and the acquired EEG data would be transmitted to the back-end computer via Bluetooth to find the spectral features by the canonical correlation analysis (CCA) algorithm. In this system, the visual stimulus device contained four flashing-frequency control commands to control the quadcopter to move up, down, forward, and turn right to the specified target. Processing EEG in the back-end computer still limited its portability. Lin et al. designed a SSVEP-based BCI system using the FPGA technique [18]. Here, the phase coding method was used as the BCI control method, and this system contained four control commands: 20 Hz–0°, 20 Hz–90°, 20 Hz–180°, and 20 Hz–270°. In this system, the FFT algorithm was implemented in FPGA to analyze EEG data and generate control commands in real time. These generated control commands would then be transmitted to the external device via Bluetooth. However, in the phase coding method, the translation time has to be extended to improve its accuracy. Unlike the above SSVEP-based BCI systems, the proposed system was designed as a wearable device. Here, active dry electrodes were designed to acquire SSVEP in hairy site without conductive gels to avoid the drying issue of conductive gels during prolonged measurements. The experimental results in Figure 8 shows that the peaks in the EEG spectra obtained by active dry electrodes exactly reflected the specific frequencies of these flashing icons. Unlike the LED-based visual stimulus used in other BCI systems, a commercial tablet was used as a visual stimulus device providing control graphic icons, and it also contained the advantages of convenience and ease of use. Moreover, in the proposed BCI system, the SSVEP BCI algorithm was implemented in FPGA for real-time computation, and this could also reduce the size of the system and allow it to be embedded into the wearable device. The experimental results showed the accuracy of the proposed system to be about 92.5%, and the averaged information transfer rate was about 36.08 (bits/min), which is higher than the 14.58 (bits/min) of FPGA-based phase coding BCI [18].

## 5. Conclusions

In this study, a wearable headset with SSVEP-based BCI was developed to acquire and translate SSVEP signals into control command directly in order to control other programs or external machines. The SSVEP BCI algorithm was also implemented in the FPGA-based BCI module to process SSVEP signal in real time. The FPGA architecture could provide the advantages of parallel signal processing to perform complex calculations in a few cycle times, and its small size also allowed it to be embedded into a wearable device. Using novel dry electrodes and a wearable mechanical design, it could easily acquire a good signal quality of SSVEP without conductive gel from the hairy site. Moreover, a portable tablet was used as a visual stimulus device in this study. The above advantages of the proposed system could effectively improve the ease and convenience of use in daily life. The experimental results showed the proposed system could effectively acquire the SSVEP signal generated by focusing on flashing control icons on the visual stimulus device and provide a good accuracy of BCI translation. The ITR of the proposed system was about 36.08 (bits/min), which is better than other BCI systems. Therefore, the proposed system can be viewed as a good system prototype of wearable BCI system, and it may be applied to various kinds of daily life applications in the future.

## Figures and Tables

**Table 1:** Comparison between the proposed system and other SSVEP-based systems.

	Lin et al. [18]	Feng et al. [30]	Wang et al. [31]	Proposed System
Accuracy (%)	95	89	83.3	92.5
ITR (bits/min)	14.58	-	4.6	36.08
Wearable system	Yes	Yes	Yes	Yes
Wireless transmission	Bluetooth	Wifi	Bluetooth	Bluetooth
Encoding	Phase coding	Frequency coding	Frequency coding	Frequency coding
Number of EEG channels	3	6	14	3
Number of control commands	4	5	4	12
EEG sensor	Wet EEG electrode	Wet EEG electrodes	Saline-based electrodes	Novel dryelectrodes
Main computing unit	FPGA	Back-end computer	Back-end computer	FPGA
Stimulus device	LED	LCD	HMD	LCD
